# Progress in Medicine

**Published:** 1899-11-25

**Authors:** 


### PROGRESS IN MEDICINE.

### NEUROLOGY.

(*Continued from page* 113.)

**Epigastric Analgesia in Tabes.**—In a healthy person a blow in the epigastic region causes an extreme degree of agony and in some cases collapse. In some pathogenic conditions, however, this is completely absent. This is often the case in hysteria, as occurred at St. Médard, where those affected with convulsions used to strike each other with stones or bars in the pit of the stomach. This epigastric analgesia is also found in some organic lesions, such as general paralysis and tabes. Pitres[10] has recently found epigastric sensation diminished in 22 out of 50 cases, and completly absent in 9. It did not appear to be accompanied by any special sensory alteration. An analogous condition is probably testicular analgesia, loss of sensation in the breast, bladder, urethra, and rectum. It is possible that all these conditions are the result of visceral neuritis, similar to the peripheral neuritis which has recently been recognised in tabes as the cause of so much alteration of special senses and general sensation.

**Acute Ataxia.**—Huyghe[11] applies the name acute ataxia to a form of tabes characterised by a very rapid onset and evolution. Difficulty in walking, the signs of Romberg, Westphal, and Argyll-Robertson are all present, together with other ocular symptoms, such as diplopia and paralysis of the third nerve. In some cases there is marked subsidence of the disease, several of the symptoms disappearing altogether, and even should some remain the condition of the patient is, generally speaking, satisfactory. The characteristic of the disease is that in the course of a couple of months the patient has reached a condition which is only attained in the ordinary form in a couple of years. As in ordinary tabes the majority of these cases are syphilitic, but, contrary to what one usually finds, purely antisyphilitic treatment is of use.

**Insular Sclerosis.**—The diagnosis of insular or disseminated sclerosis from hysteria, more particularly in the early stages, is sometimes a matter of the greatest difficulty. We owe to Buzzard[12] much of our knowledge of the means of differentiation. He points out that insular sclerosis is most commonly ushered in by weakness in one of the lower extremities, often of sudden onset, perhaps accompanied or preceded by a feeling of numbness. On the other hand, hysterical loss of power is likely to be more pronounced than that which attends the beginning of insular sclerosis, and to be accompanied with more marked sensory derangement, especially anæsthesia. Temporary recovery is more usual in cases of insular sclerosis than in hysteria. Transitory convergent strabismus is also indicative of sclerosis rather than of hysteria; so also is amblyopia, especially if associated with pallor of the optic disk. The knee jerks may be increased in hysteria, but they are never lost, and there is never persistent or well-marked ankle-clonus. Intention tremor, nystagmus, and scanning speech are never found in hysteria. Difficulty in the expulsion of urine may be found both in hysteria and insular sclerosis, but in the latter it may go on to incontinence. Anæsthesia of the legs is a symptom of hysterical paraplegia rather than of insular sclerosis. Hysterical contracture of the legs differs from the contracture of insular sclerosis by being associated with well-marked and persistent ankle-clonus, and is usually sudden in onset, whilst that of insular sclerosis is a gradual progress. The plantar reflex, referred to above, is usually lost in hysterical paraplegia; it may be absent in insular sclerosis also, but the associated symptoms will aid in the diagnosis. The extensor type of plantar reflex is never found in simple hysteria; it invariably denotes involvement of the pyramidal tract by an organic lesion.

**Pulse and Temperature in Epilepsy and Hystero-epilepsy.**—Marchand[13] has made a clinical study on pulse and temperature in 300 cases observed in St. Anne's Asylum. He finds that the epileptic attack causes an elevation of internal body temperature of ·5 deg. C., lasting on an average 40 minutes. Attacks of epileptic vertigo cause a less marked but still noticeable rise of temperature. The epileptiform attacks of general paralysis of the insane cause a decided elevation of temperature comparable to that of true epilepsy. There is in general no relation between the age of the patient and the maximum of temperature, and in the same patient different attacks may cause various temperatures. The pulse rises about 30 beats above the normal in a fit, but has fallen to normal within an hour; the maximum rate is 15 minutes after an attack. Both pulse and temperature return to the normal steadily or with only slight oscillations. In epileptic vertigo the pulse also rises in proportion to the intensity of the attacks. In hystero-epilepsy the temperature rises, and there is a relation between the length of the attack and the elevation of temperature. The maximum frequency of the pulse in hystero-epilepsy is at the onset of the attack (epileptoid period). On the whole the pulse disturbances are much more marked than the temperature changes in hystero-epilepsy.

**Muscular Hypotonia in Epileptics.**—Rennie[14] has described cases of muscular hypotonia as an interparoxysmal condition in epileptics. In normal conditions the muscles are kept in a condition of slight tension, which is maintained in whatever state they may be in, whether at rest or acting with other muscles. This muscle tonus is maintained by some reflex influence. Thus, if the posterior roots be cut experimentally the tonus of the corresponding muscles ceases. Such a condition occurs in tabes and in Friedreich's ataxy, owing to the loss of afferent muscle impressions caused by the disease of the posterior roots. In epileptics, with frequently recurring fits, a condition of lessened tone of the muscles may become a more or less permanent interparoxysmal phenomenon. It is due to a state of exhaustion of the motor cortical cells.

**Epilepsy.**—Hester[15] has made experiments on the toxicity of the urine and blood in cases of epilepsy. Haig has claimed that uric acid was the actual poison causing the epileptic paroxysms, but it has never been shown that uric acid, in the quantity in which it existed in the body, was a poison. The French had made much of the toxicity of the urine in connection with the subject of epilepsy. Their experiments had been based on the introduction of urine into rabbits, but this method was exceedingly inaccurate, because the toxicity of urine depended very largely upon the quantity of mineral salts present, and unless the quantity of these substances present was known nothing definite could be said about the toxicity of urine. The most interesting phase of the subject was that dealing with the toxicity of the blood, and its relation to epileptic seizures. It was only by the greatest care that reliable results could be obtained from the experimental injection of serum into rabbits. He had examined 18 cases of epilepsy, and could say that in 15 of these there had not been any special toxicity of the blood.

**Bromide of Camphor in Epilepsy.**—Haste[16] found that, as regards epilepsy proper (haut mal), the action of bromide of camphor was doubtful, and was less effectual than the mixed bromides of potassium, sodium, and ammonium. But in attacks of petit mal, and in all cases of epileptic vertigo, its effect was incontestable. It at first diminished the frequency of the attacks, and finally made them disappear altogether. The drug was prescribed in moderate doses, 40 centigrammes, gradually increased to 160 centigrammes, and then gradually diminished until 40 centigrammes was reached, and this last dose was maintained for some time.

**Bromide of Strontium in Epilepsy.**—Smith[17] has recently compared the action of bromides of strontium and potassium in controlling epileptic fits. He finds that there was only a slight difference between the effects of the two drugs, though what difference there was was clearly in favour of the strontium salts as regards the absolute number of fits. A larger dose of bromide of strontium was required to control the fits than of the potassium salt, in the proportion of 3 to 2. The bromide of potassium seemed to exercise its action more quickly than the strontium bromide. Lastly, the good effect of the bromide of potassium seemed to be more lasting than that of bromide of strontium, as it was found necessary to increase the dose of the latter at shorter intervals. Hence, on account of the more rapid action, more lasting effect, smaller dose required, and greater cheapness, the potassium salt is to be preferred.

[10] Pitres, Jour. de Méd., Jan., 1899. [11] Huyghe, Thèse de Lyon, 1898. [12] Buzzard, Brit. Med. Jour., May 6, 1899. [13] Marchand, Thèse de Paris, 1899. [14] Rennie, Lancet, July 15, 1899. [15] Hester, Med. Rec., May 20, 1899. [16] Hasté, Thèse de Paris, 1899. [17] Smith, Lancet, Aug. 12, 1899.

### DISEASES OF THE DIGESTIVE ORGANS.

(*Continued from page* 115.)

**Small Intestines.**—Burney Yeo[42] discusses a curious case of chronic diarrhœa where no organic disease could be detected. The stools were very light coloured, often five or six in number in 24 hours, and the patient was losing ground rapidly. He ordered complete rest and pure milk diet, with a teaspoonful of pancreatic emulsion twice a day. In addition he administered ten drops of tincture of coto bark in a bismuth mixture. The milk was gradually increased, and cream, meat juice, and eggs added. The patient put on flesh, and made a good recovery after three years' suffering. He suggests that the failure of the pancreas in such cases to digest fats is a possible cause of the irritation. Richter,[43] in the treatment of catarrh and ulceration of the intestine, gives certain astringents before breakfast, and at the same time washes out the large intestines. The drug thus passes along an empty bowel, and, indeed, a dose of castor oil may be given beforehand to effect this purpose thoroughly. Tannin bismuth and a decoction of bilberries are especially serviceable when employed in this manner. No food should be taken for some time afterwards, and a second dose may precede the evening meal. Vomiting of enemata has been recorded in some three instances, and appears to be a definite fact. Parkes Weber[44] found that oil and methylene blue were vomited some ten minutes after injection into the rectum. Laparotomy showed that there was no fistula from the colon into the stomach, and in another case the ileocæcal valve proved to be normal. Weber thinks that a spasmodic contraction occurs in the lower end of the bowel which acts like a stricture, setting up reversed peristaltic action in the intestines generally. The mode of intestinal absorption has been investigated by Weymouth Reid,[45] who lays stress on the activity of the epithelium. If this is removed or destroyed the quantity taken up is reduced, or even done away with altogether. The cells absorb most rapidly salts, next water, and last, organic solids, and the activity of the ileum is greater than that of an equal area of the colon. Corlette shows that excretion also takes place into the small intestine. The substances excreted contain free fatty acids, soaps, and cholesterin, but no urobilin or hæmoglobin.

B. Nesbitt[46] proves that when the small intestine is completely occluded neurin and cholin are soon produced if the food contains much lecithin. Other poisons may be formed by bacterial action as well. Neurin, however, is akin to muscarin, and its action on the heart is rapid. Hence, we should avoid all such food as eggs, which contain much lecithin.

**Dysentery.**—A. Hillier[47] recommends for the treatment of chronic dysentery frequent small doses of castor oil and opium. Thus, he usually gives one or two drachms of the former with five to ten minims of laudanum thrice daily, while the diet is restricted to milk and broth, or even whey. Large enemata of warm milk, after Dr. Jameson's practice, are found most useful and soothing. Injections of quinine and of nitrate of silver have been also recommended by other writers. W. Stewart maintains that when the disease arises from hepatic congestion relief is best given by chloride of ammonium. In other forms he prefers ipecacuanha in full doses.

**Intestinal Astringents.**—A great number of new astringents have been brought forward. We may notice the following amongst others: Tannigen, which is soluble only in alkalies, and therefore passes through the stomach without irritation, has been recommended for diarrhœa due to intestinal catarrh by several authorities. Ewald[48] speaks highly of it for children after a dose of castor oil. Others recommend it in three to ten grain doses for adults in chronic cases. Moncorvo found it of value in malarial and phthisical patients. In tannopine we have a combination of tannic acid and urotropine, with the astringent and antiseptic effects of both elements. In tubercular and other diarrhœas, both acute and chronic, it has given good results in many cases under Schreiber, Fuchs, and Meici. Tannocol, a mixture of tannin and gelatine, which also passes unchanged through the stomach, is praised by Goliner,[49] who found it useful in 15 grain doses for adults and in reduced amounts for children, with tubercular and other diarrhœas. Tannoform, another combination with formaldehyde, has been employed not only for diarrhœa, but also in diseases of the nose, throat, uterus, and skin diseases, especially eczema and hyper-idriosis. St. Grosse[50] gives an interesting summary of the various foreign papers which have appeared on its applications. It is given in 15 grain doses with excellent effects, both astringent and antiseptic, in acute and chronic catarrhs of the intestines. The estimation of indican as a measure of fermentation in the intestine is of some importance, and it may be worth recording Volowsky's simple process.[51] He first treats the urine with acetate of lead and filters. Five c.c. are placed in each of three test tubes, and one, two, and three drops respectively of a solution of chlorinated potash are added and the tubes inverted several times. Five c.c. of H.cl. are then added and the tubes cooled, and finally 1 c.c. of chloroform is poured in and the tube inverted several times quietly. If the indican is only normal—about ·006 grammes in the 24 hours' urine—the chloroform will be slightly coloured in the first tube, more so in the second, and clear in the third. If there is an excess more drops are added till the colour vanishes. The amount causing the deepest tint is noted, and an easy calculation gives the quantity of indican in 24 hours' urine, as each drop of the solution represents ·1 gramme of chlorine per litre.

**Rashes after Enemata** are recorded in six cases by Monro.[52] They may occur from two to twenty hours or longer after the injection, and last usually two or three days. In form they may be measly, urticarial, or like scarlatina. There may or may not be itching, and very rarely a rise of temperature. Coupland once noticed desquamation. On one theory they are due to fæcal absorption, but there is also reason to think that they depend on the use of hard yellow soap. However, it is only after bulky enemata that they appear, never apparently after glycerine or nutrient ones, and second injections of the same kind do not always produce them in susceptible persons.

42 Lancet, July 22. 43 Brit. Med. Jour., June 10. 44 Practitioner, Aug. 45 Brit. Med. Jour., July 22. 46 Jour. of Exp. Med. Vol. IV. 47 Indian Med. Rec., April 26. 48 Clinical Excepts, June. 49 Therapist, May. 50 Les Nouv. Remèdes, Sept. 8. 51 Internat. Med. Mag., Aug. 52 Glasgow Med. Jour., Sept. 53 Lancet, Sept. 23.

